# A century of “anticoccidial drugs”: bibliometric analysis

**DOI:** 10.3389/fvets.2023.1157683

**Published:** 2023-05-02

**Authors:** Mahmoud Kandeel, Mohamed A. Morsy, Hany M. Abd El-Lateef, Mohamed Marzok, Hossam S. El-Beltagi, Khalid M. Al Khodair, Ibrahim Albokhadaim, Katharigatta N. Venugopala, Mohammed Al-Rasheed, Mahmoud M. Ismail

**Affiliations:** ^1^Department of Biomedical Sciences, College of Veterinary Medicine, King Faisal University, Al-Ahsa, Saudi Arabia; ^2^Department of Pharmacology, Faculty of Veterinary Medicine, Kafrelsheikh University, Kafrelsheikh, Egypt; ^3^Department of Pharmaceutical Sciences, College of Clinical Pharmacy, King Faisal University, Al-Ahsa, Saudi Arabia; ^4^Department of Pharmacology, Faculty of Medicine, Minia University, El-Minia, Egypt; ^5^Department of Chemistry, College of Science, King Faisal University, Al-Ahsa, Saudi Arabia; ^6^Department of Chemistry, Faculty of Science, Sohag University, Sohag, Egypt; ^7^Department of Clinical Sciences, College of Veterinary Medicine, King Faisal University, Al-Ahsa, Saudi Arabia; ^8^Department of Surgery, Faculty of Veterinary Medicine, Kafrelsheikh University, Kafrelsheikh, Egypt; ^9^Agricultural Biotechnology Department, College of Agriculture and Food Sciences, King Faisal University, Al-Ahsa, Saudi Arabia; ^10^Biochemistry Department, Faculty of Agriculture, Cairo University, Giza, Egypt; ^11^Department of Anatomy, College of Veterinary Medicine, King Faisal University, Al-Ahsa, Saudi Arabia; ^12^Department of Biotechnology and Food Science, Faculty of Applied Sciences, Durban University of Technology, Durban, South Africa; ^13^College of Veterinary Medicine, Avian Research Center, King Faisal University, Al-Ahsa, Saudi Arabia; ^14^Department of Poultry and Rabbit Diseases, Faculty of Veterinary Medicine, Kafrelsheikh University, Kafrelsheikh, Egypt

**Keywords:** anticoccidial, bibliometric, research, publications, analysis

## Abstract

Publications are an important measure of scientific and technological progress. The quantitative examination of the number of publications in a certain research topic is known as bibliometrics. Bibliographic studies are widely used to analyse the condition of research, future potential, and current growth patterns in a certain topic. It can serve as a basis for making decisions and implementing strategies to achieve long-term development goals. To our knowledge, no research has been conducted in these domains; so, this work aims to employ bibliometric analysis to provide comprehensive data on publications related to anticoccidial drugs. As a result, the current study uses bibliometric analysis to track the evolution of anticoccidial drugs and its consequences in the academic and public worlds via a survey of relevant scientific and popular publications. The Dimensions database was used to retrieve the bibliographical statistics, which were then cleaned and analyzed. The data was also loaded into the VOS viewer, which generated a network visualization of the authors with the most joint articles. The investigation discovered three stages of publications and citations since the first article on anticoccidial drugs in 1949. The first stage, which ran from 1920 to 1968, was characterized by a scarcity of research articles on anticoccidial drugs. From 1969 to 2000, the second stage was marked by a stable and marginally increased number of articles. The scientific field was characterized by an increasing trend in the number of publications and their citations from 2002 to 2021. The study gave a complete list of the top anticoccidial drugs funding agents, countries, research institutes, most cited publications, and important co-authorship and partnerships. The outcomes of the study will help veterinary practitioners and researchers understand the trends and best sources of knowledge in the field of anticoccidial medications.

## 1. Introduction

Over the past several decades, numerous attempts have been conducted to unpack and dissect anticoccidial drugs’ development, efficacy, and challenges. Examples of anticoccidial drug subgroups include Ionophores, Sulphonamides, Vitamin Antagonists, Nicarbazin, Quinolones, and others (1). Anticoccidial drugs are used to treat infectious diseases in the intestinal tract along with the new development in coccidiosis vaccines (2, 3). An ideal anticoccidial drug has been found to have high efficacy in performing a broad-spectrum activity (4). In addition, an ideal drug must have large therapeutic index (TI). A drug with a high therapeutic index is considered safer than one with a low therapeutic index because it can be administered at a wider range of doses before adverse effects occur. The drug is also should be cost-effective, making it adequately affordable to the market (5). Besides, some past researchers have observed that most drug subgroups do not affect organoleptic criteria in determining the meat and carcass quality. Ideally, most anticoccidial drugs can be metabolized and excreted without toxic residuals (6).

Coccidiostat failure has also been observed in several publications. Some of the causes identified include more than average oocysts exposure in the host. Besides, some of the drug subgroups may not be effective when treating or preventing all the Eimeria species (7–9). Besides, poor management, especially in poultry production, has also been identified as a cause of wet litter, increasing the risk of Coccidiostat failure (10). Prolonged drug use and intercurrent diseases can lead to drug resistance (7, 11). The efficacy of the anticoccidial drugs has been discussed widely and in-depth, with numerous publications being conducted since 1955 when Nicarbazin, the first broad-spectrum anticoccidial drug was approved (2, 12–14).

The study will employ prior literature as datasets holding information such as authors, titles of publications, years of publication, funding agents, the source institutes and countries. As a result, these data be used to establish a quantitative intellectual framework for anticoccidial medication research. However, due to the enormous number of publications on the subject, manually gathering and compiling all data is impractical, thanks to internet databases and software-based techniques that can easily enable effective data accessibility. As a result, the study seeks to give a bibliometric and visual analysis of anticoccidial drugs by studying the intellectual domain and the time evolution of anticoccidial drug research.

The objectives of this study are 1) Analyze the trend in anticoccidial drugs publications in terms of the volume of documentation and citations in the field, 2) Analyze the most frequently cited research to contribute to the knowledge in the field of anticoccidial drugs, 3) Create awareness of the topmost recognized publishers, the most active country, the most active institutions and the top most funding agents in the field of anticoccidial drugs, and 4) Highlight the topmost authors with the highest number of jointly authored publications on anticoccidial drugs. To the best of our knowledge, no research has been done on these areas of thought, hence the goal of this paper is to use bibliometric analysis to give full data on anticoccidial drug publications.

## 2. Materials and methods

### 2.1. Research questions

The questions raised in this study include 1) What is the trend in the volume of publications and documentation citation of the anticoccidial drugs over the past century? 2) What are the topmost countries that researchers prefer for their works on anticoccidial drugs? 3) What are the topmost researchers, research institutes, and frequent funding agents in the field of anticoccidial drugs? 4) What is the intellectual structure of the joint authorship on anticoccidial drug publications?

### 2.2. Bibliometric approach

The research employs bibliometric analysis and science mapping to perform the quantitative and visual analysis of anticoccidial drugs. Bibliometrics was first created by Otlet in 1934 (15). Later, Broadus defined the term as the quantitative study of bibliographic units or published units (16). In 1969, Pritchard coined Bibliometrics to replace the statistical bibliography (17). The research approach has become a valuable tool for evaluating the research outputs and dealing with ever-increasing information. Besides, databases and software have made it possible and practical to obtain and analyze massive and complex bibliometric data (18). Currently, several tools exist to analyze the trends of publications, visualize the citation network, and identify new topics and trend patterns for the scientific disciplines. These tools can conduct a science mapping using the bibliometric approach to produce a spatial representation of networks. The information from the publications, such as the publication’s year, title, author(s), and citations, are crucial in providing these visual representations (19). Besides, the visual maps allow one to view the linkages and development of knowledge that would not be possible without statistics.

For this study, data collection involves collecting relevant literature published and collected in different databases for further analysis. The data collection was achieved by first identifying the databases and choosing appropriate search strategy techniques, data retrieval techniques, and cleaning the data before feeding them into different tools for analysis and visualization.

### 2.3. Retrieval of data

Nowadays, several databases exist that offer bibliometric data. Examples of such databases include Web of Science, Scopus, PubMed, Cochrane Central Register of Controlled Trials (CENTRAL), Google Scholar, Dimensions, CORE, BASE, Science.gov, Crossref API, Microsoft Academic Search, and JSTOR Data for Research. For this research, Dimensions^1^ will be used to retrieve the bibliometric data because it is open software, and the data will be easily integrated into the VOS viewer. The study will use VOS viewer as the software tool to provide the network visualizations for the bibliometric data on anticoccidial drugs. VOS is a free online computer program for scientific mapping, offering a cluster display of complex networks (20).

In this study, the “dimensions database” was used to retrieve bibliometric data owing to 1) Dimensions covers a wider range of research outputs beyond scholarly articles and conference proceedings, including grants, datasets, clinical trials, patents, and policy documents, 2) citation analysis tools that allow users to identify highly cited articles and authors, track citation trends over time, and measure the impact of research, and 3) Dimensions has a more modern and intuitive user interface that allows users to filter and sort search results more easily. It also offers more visualization tools and interactive features, such as citation maps and co-author networks.

### 2.4. Search strategy, exclusion criteria and limitations

The study used “anticoccidial drug” as the keyword to search relevant publications. The data retrieved comprised all the publications with the term “anticoccidial drug” in either their title, abstract or the main document. The study did not exclude any of the parameters provided in the dimensions database since the study intended to explore all the publications stored on anticoccidial drugs. The documents retrieved included articles, book chapters, editorial materials, and reviews. The search strategy retrieved a total of 5,000 publications. However, using a single dataset was limiting since some critical publications may not have been included in the Dimensions Database. Besides, the database also does not provide some bibliometric features such as keywords and references compared to other Databases. Since only English words were employed to search for relevant publications, the search may have left out some critical articles published in other languages such as French, Spanish, and Chinese. Therefore, the results may not be generalized to other researchers published in non-English speaking countries. Although the analysis may not include all the crucial publications on anticoccidial drugs, this study’s results offer a reliable insight into the trends and patterns in the publications that have been made on anticoccidial drugs.

### 2.5. Preferred reporting items for systematic reviews and meta-analysis (PRISMA)

**Figure 1:**
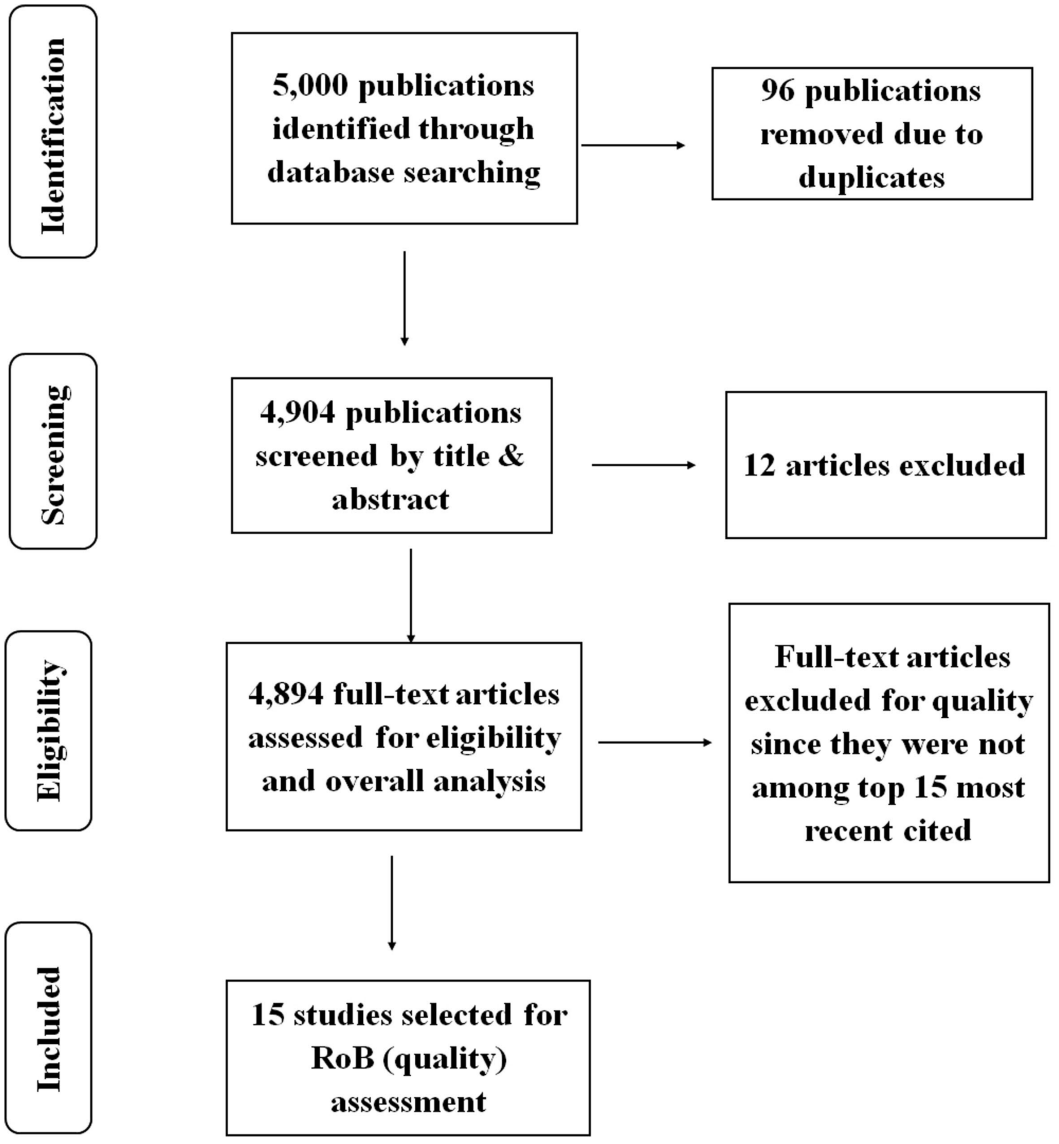
PRISMA flow chart for bibliometric analysis of anticoccidial drugs.

The study employed PRISMA in identifying, screening, and selecting the studies to be included in various analyses. The criteria were inclusive of all the years since the trend analysis was crucial in determining the progress made in terms of publications. Although the criteria did not specify a particular type of study, the database selection generated articles, chapters, edited books, and reprinted publications only. Upon retrieving the publications’ data, the data was cleaned by removing duplicates. The study also excluded publications that were not relevant to the study. Only the publications with the most recent citations were included in the risk of bias assessment. Details of the screening process and selection of the studies are shown in Figure 1.

### 2.6. Quality assessment and risk of bias

Two independent researchers assessed the selected publications to determine their eligibility for the study. The majority of the selected articles were considered to be of high quality in terms of the methodology used and the relevance of the abstract to the study’s issue. During the screening stage for each study, the researchers were keen to determine the method of study participant selection, the strategy to reporting used, the methodologies used to measure the outcomes, the presence of detection bias, and attrition bias. Depending on the information given in the publications, each component was classified as uncertain, low, or high. Summary of quality assessment outcomes is provided in Table 1.

**Table 1:** Quality assessment for risk of systematic bias for the articles included in the systematic review on the efficacy of anticoccidial drugs (categorized as low, high, and unclear).

Study (author and year)		Selection of participants (Selection bias)	Selective outcome reporting	Measurement of exposure (measurement exposure)	Blinding of outcome assessment (detection bias)	Incomplete outcome data (attrition bias)
1	Johnson and Reid (21)	unclear	low	high	unclear	low
2	Clavijo and Flórez (22)	high	low	unclear	unclear	low
3	Dalloul and Lillehoj (3)	low	low	low	high	low
4	Blake and Tomley (8)	unclear	low	high	low	low
5	Williams (23)	high	low	low	unclear	low
6	Danzeisen et al. (24)	low	high	low	unclear	unclear
7	Chapman (25)	unclear	unclear	low	unclear	low
8	Peek and Landman (26)	low	unclear	high	low	low
9	Blake et al. (27)	high	low	unclear	low	unclear
10	Chapman et al. (28)	low	high	unclear	unclear	low
11	Noack et al. (2)	high	high	high	unclear	low
12	Quiroz-Castañeda and Dantán-González (29)	low	unclear	unclear	low	high
13	Williams (30)	high	low	low	unclear	unclear
14	Collier et al. (9)	unclear	low	high	high	unclear
15	Chapman et al. (31)	unclear	low	high	low	low

## 3. Results

### 3.1. Bibliometric analysis of the publication trends in anticoccidial drugs

**Figure 2:**
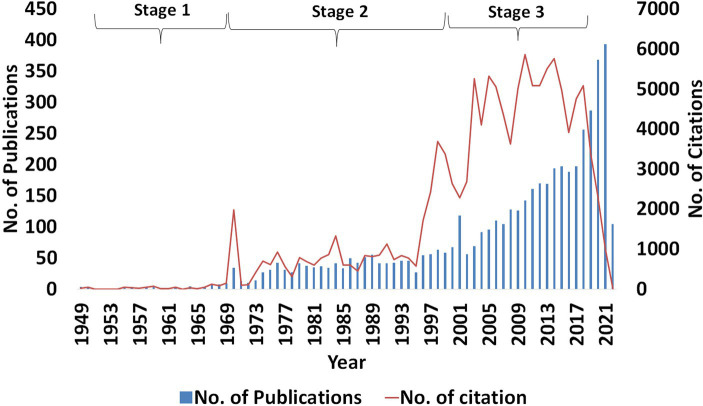
Annual total output of publications and citations on anticoccidial drugs. The data was retrieved from 1920 to 2021.

The bibliometric study on anticoccidial drugs from 1920 to 2021 revealed three stages of development based on the total publication in each of the years, as shown in Figure 2. The first stage, identified from 1920 to 1968, was marked by a low number of research publications related to anticoccidial drugs. This was likely because the research was not fully developed in that period. The second stage, which runs from 1969 to 2000, was marked by constant and slightly significant publications until 2001 when there was a surge in the number of publications, almost double from the previous year. The third stage began in 2002 to 2021, where the research field was marked by an ever-constant increasing trend in the number of publications and their citations. The number of citations in the two last years has decreased because the recent publications did not have the advantage of the time to allow awareness among the researchers, pending inclusion into the database and the closure due to COVID-19.

### 3.2. Contribution to the anticoccidial field by country

**Figure 3:**
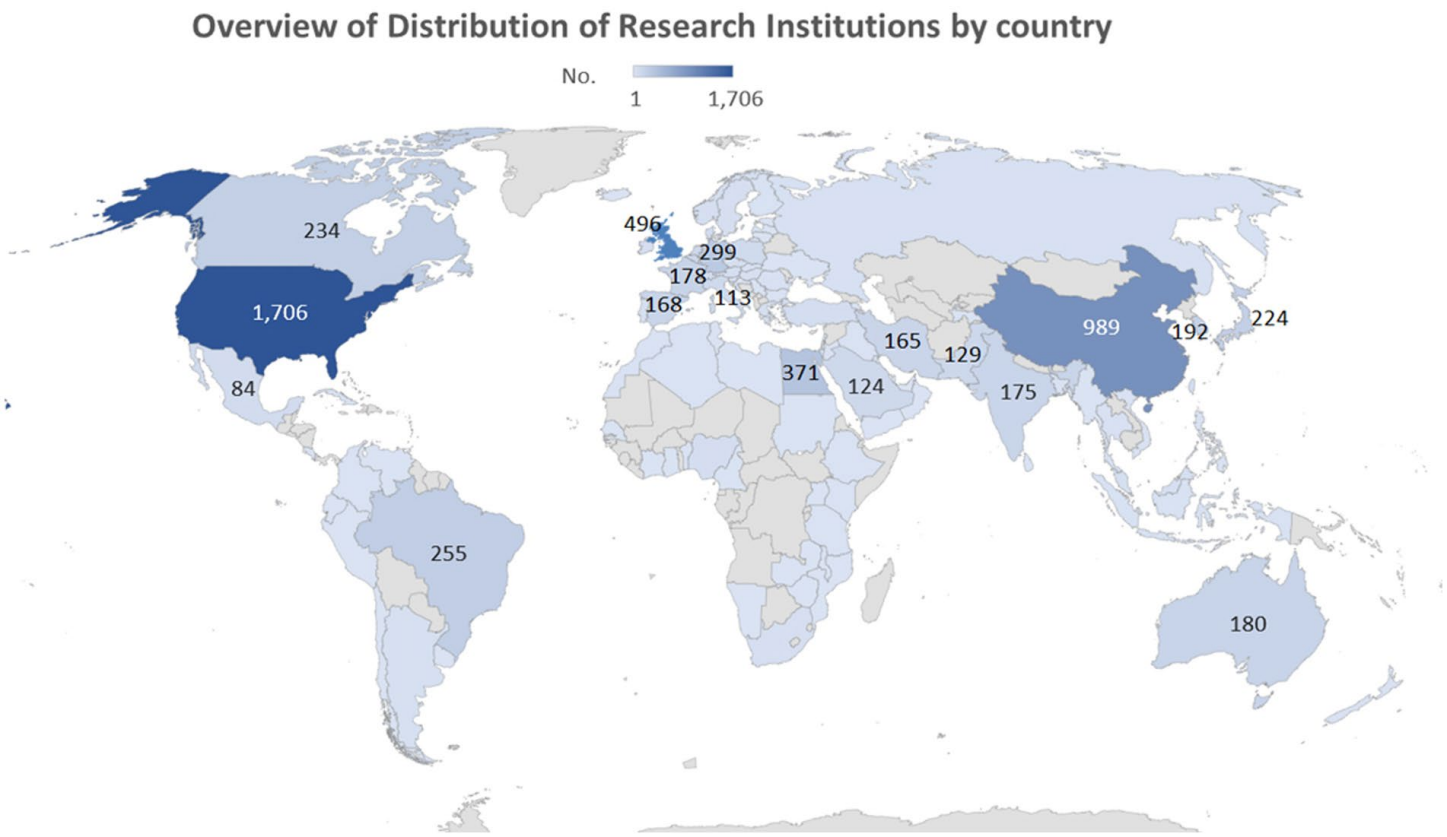
Overview of Distribution of Countries with more than 100 publications on anticoccidial drugs.

**Figure 4:**
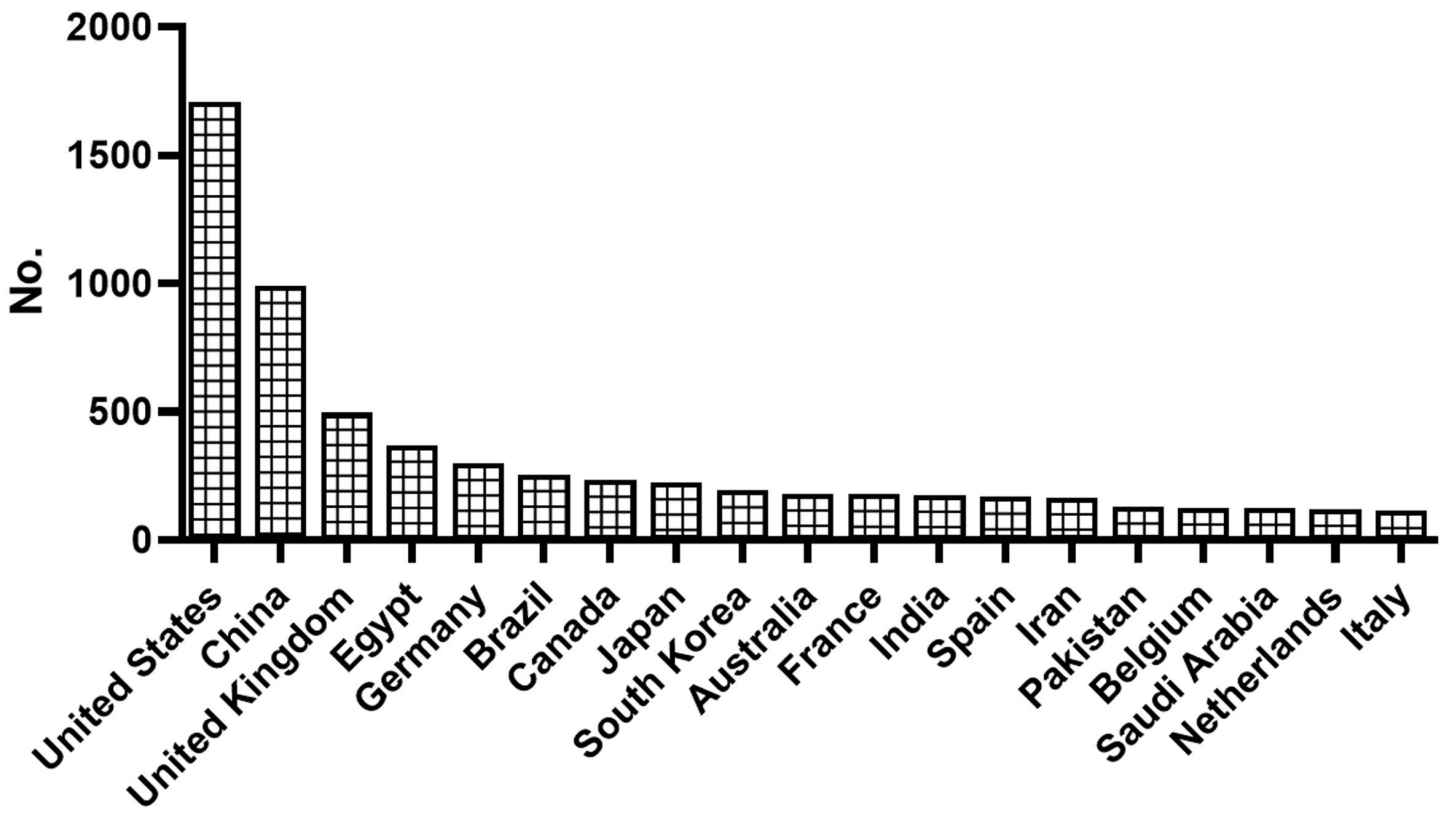
The rank of countries with more than 100 publications in the field of anticoccidial drugs.

The study revealed that publications on anticoccidial drugs originated from 102 countries. The United States was identified as the leading country of publications, followed by China and the United Kingdom. Figures 3, 4 show the overall distribution of the countries and the countries with over 100 publications, respectively. The full list of per-country publications is provided in Supplementary Table S1.

### 3.3. The leading institutions

**Figure 5:**
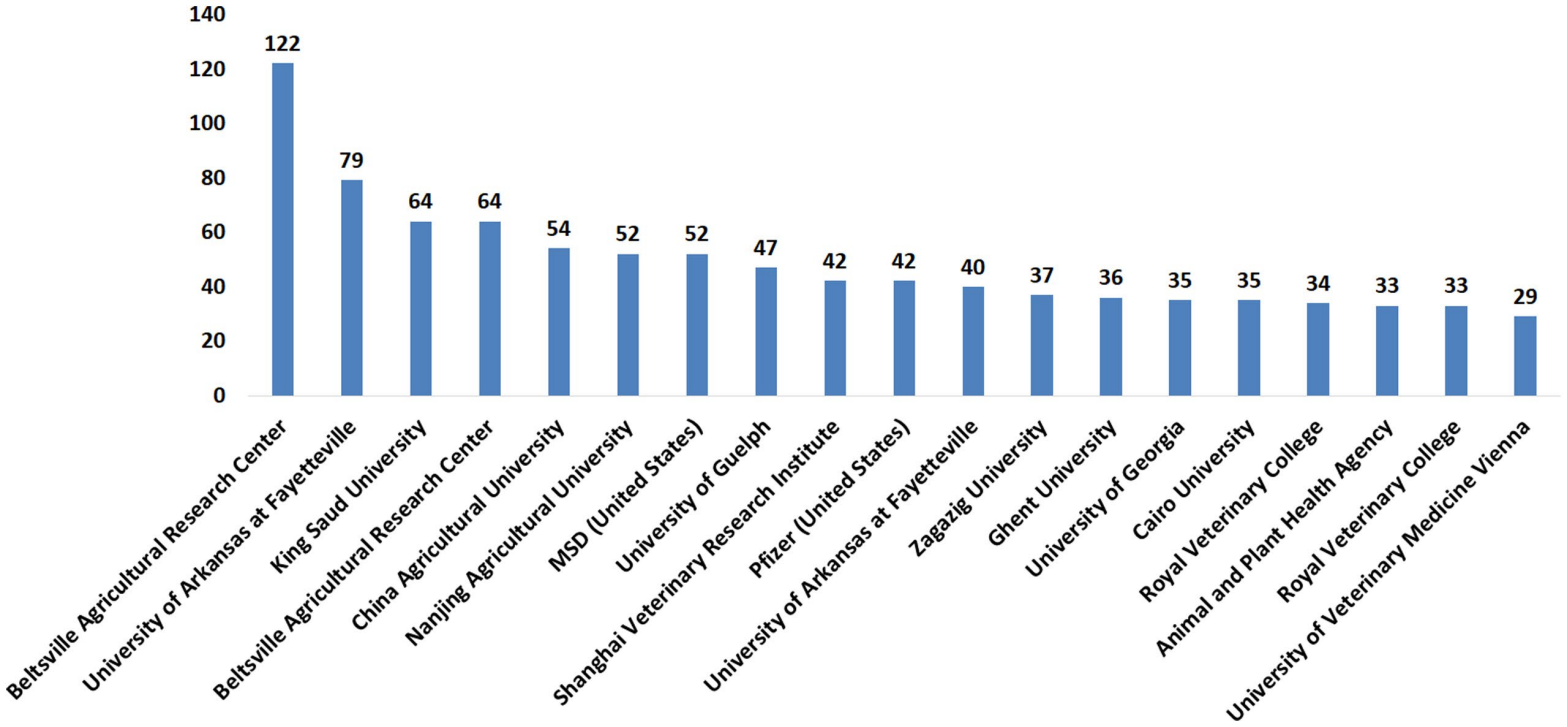
The Research Institutions with over 30 publications on anticoccidial drugs.

The publications on anticoccidial drugs were investigated by the contribution of research institutes. The leading research institutions with more than 30 publications evaluating the anticoccidial drug are provided in Figure 5. The full list of per-institute publications is provided in Supplementary Table S2. These institutions contributed to the previously described country’s rank with the top four countries in anticoccidial publications, United States, United Kingdom, China, and Egypt.

### 3.4. The most frequent funding agents

**Figure 6:**
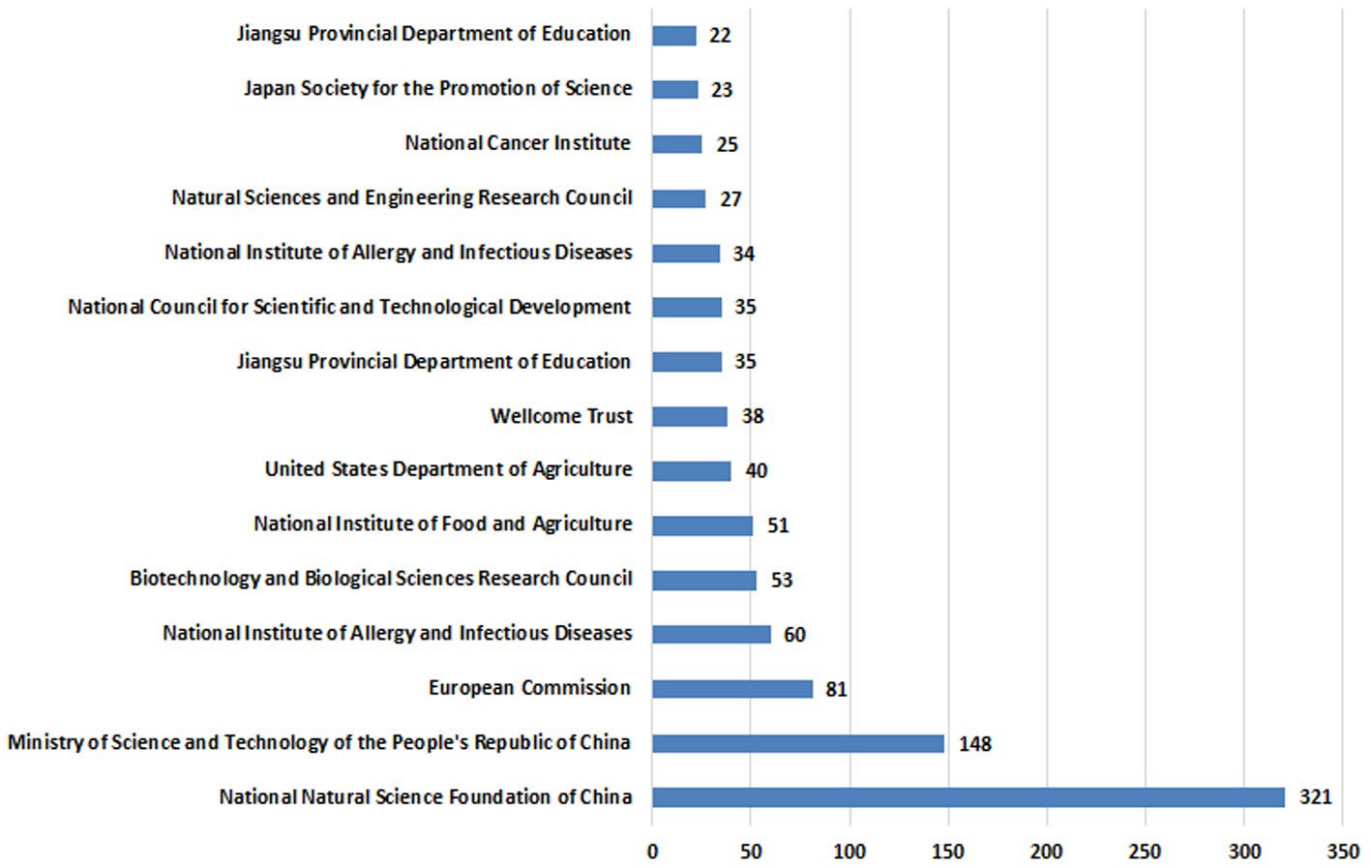
The Most Frequent Funding Agents for anticoccidial drugs.

The most frequent funding agents are displayed in Figure 6 and Supplementary Table S3. The National Natural Science Foundation of China is the leading funding institution in this field, having funded 312 research. Among the top funding agents was the European commission, US dept. of Agriculture, Japan JSPS and the Australian research council.

### 3.5. The recently cited publications on anticoccidial drugs

In terms of the most widely recently cited publications on anticoccidial drugs, Johnson & Reid (21) lead both in the total citations as well as the most recently cited works despite having made the publication in 1970. The experiments on chicken lesion scoring seem to have remained constant throughout the years and are widely recognized by researchers in this field. A study on gastrointestinal microbiome among broiler chickens published just recently in 2017 (22) was in the second rank with 149 recent citations despite having been published less than 50 years ago. Among the 15 selected research on anticoccidial drugs, all of them have more than 50 recent citations, revealing that they have received widespread interest in this field (Table 2).

**Table 2:** Most recently cited papers in anticoccidial drugs.

*R*	Total citation	Title	Authors	Year	Recent citations	Reference
1	876	Anticoccidial drugs: Lesion scoring techniques in battery and floor-pen experiments with chickens	Johnson and Reid	1970	159	(21)
2	190	The gastrointestinal microbiome and its association with the control of pathogens in broiler chicken production: A review	Clavijo and Flórez	2017	142	(22)
3	364	Poultry coccidiosis: recent advancements in control measures and vaccine development	Dalloul and Lillehoj	2006	98	(3)
4	230	Securing poultry production from the ever-present Eimeria challenge	Blake and Tomley	2013	98	(8)
5	319	Intercurrent coccidiosis and necrotic enteritis of chickens: rational, integrated disease management by maintenance of gut integrity	Williams	2005	96	(23)
6	244	Modulations of the Chicken Cecal Microbiome and Metagenome in Response to Anticoccidial and Growth Promoter Treatment	Danzeisen et al.	2011	82	(24)
7	167	Milestones in avian coccidiosis research: A review	Chapman	2014	82	(25)
8	183	Coccidiosis in poultry: anticoccidial products, vaccines, and other prevention strategies	Peek and Landman	2011	79	(26)
9	67	Re-calculating the cost of coccidiosis in chickens	Blake et al.	2020	67	(27)
10	177	Forty years of monensin for the control of coccidiosis in poultry	Chapman et al.	2010	63	(28)
11	63	Anticoccidial drugs of the livestock industry	Noack et al.	2019	61	(2)
12	96	Control of Avian Coccidiosis: Future and Present Natural Alternatives	Quiroz-Castañeda and Dantán-González	2015	60	(29)
13	298	A compartmentalised model for the estimation of the cost of coccidiosis to the world’s chicken production industry	Williams	1999	59	(30)
14	197	Coccidia-induced mucogenesis promotes the onset of necrotic enteritis by supporting Clostridium perfringens growth	Collier et al.	2007	55	(9)
15	140	A Selective Review of Advances in Coccidiosis Research	Chapman et al.	2013	51	(31)

### 3.6. Topmost authors with joint publications

**Figure 7:**
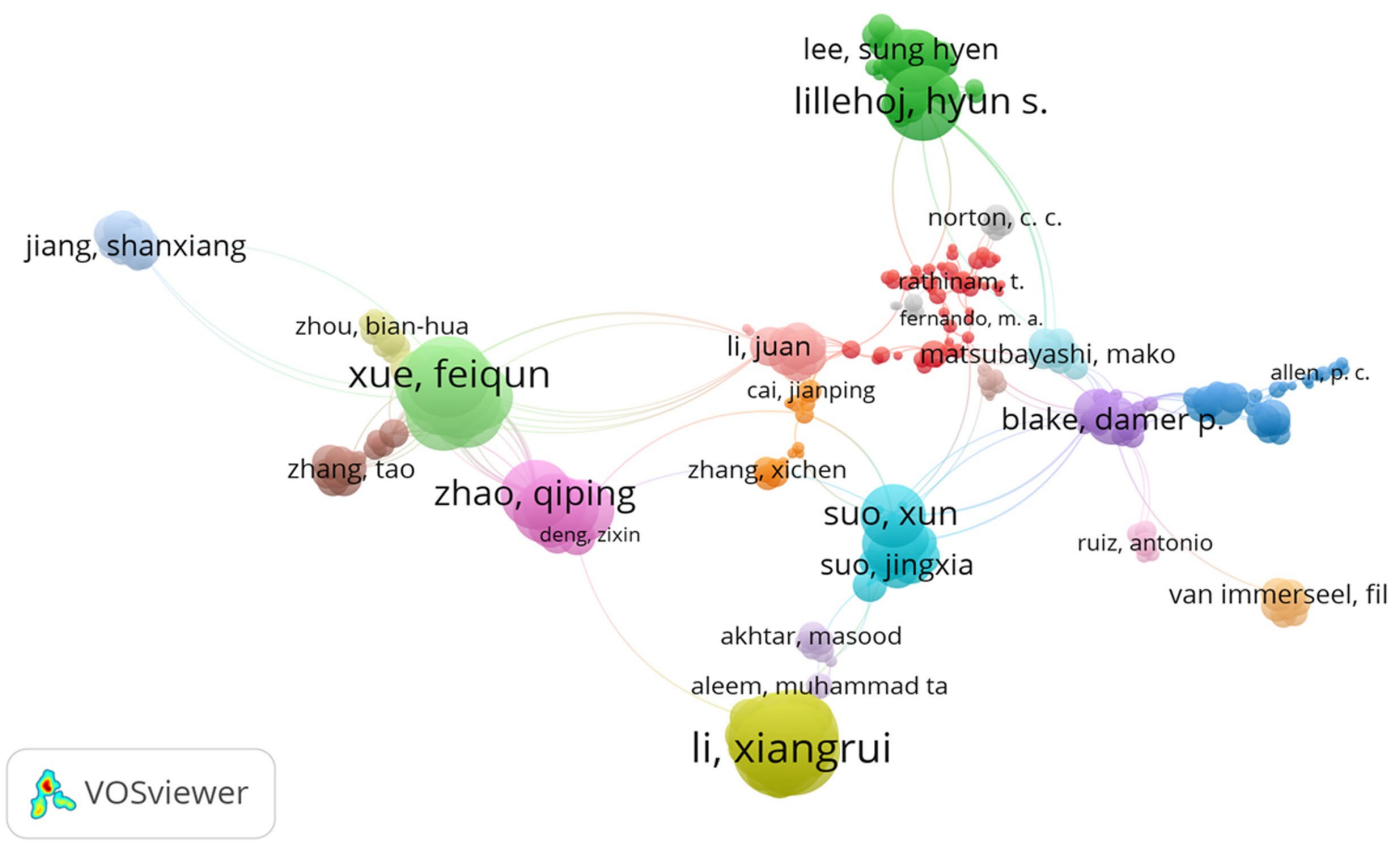
Intellectual structure of the topmost authors with joint publications.

The study used the VOS viewer to visualize the authors with the highest collaboration in anticoccidial drugs. Based on the network visualization provided in Figure 7, Li Xiangrui, Zhao Qiping, Suo Xun and Lillehoj Hyun were among the highest authors in anticoccidial drugs with the most joint publications. The network helps identify the authors with the highest collaborations and shows that authors can jointly publish with other authors. The different colours identify the various topics discussed by the authors related to anticoccidial drugs.

### 3.7. Eimeria species

The fraction of publications citing Eimeria species was traced in the publications. The traced species comprise *Eimeria acervulina, Eimeria brunetti, Eimeria maxima, Eimeria mitis, Eimeria necatrix, Eimeria praecox*, and *Eimeria tenella*. Each species of Eimeria affects a different part of the chicken’s digestive tract, and can cause a range of symptoms including diarrhea, decreased appetite, weight loss, and even death (32). The search comprised the inclusion of species name in the title or abstract of the publication. The outcomes of the search revealed 1,073 publications for *E. acervulina*, 3,854 for *E. brunetti*, 10,964 for *E. maxima*, 3,189 for *E. mitis*, 5,840 for *E. necatrix*, 3,414 for *E. praecox*, and 18,971 *E. tenella*.

In comparison with the chicken Eimeria species, the bovine and ovine Eimeria also received a great bulk of scientific contributions. The retrieved numbers were 12,090 for *E. bovis*, 3,226 for *E. ellipsoidalis*, 3,236 for *E. cylindrica* and 3,611 for E. zuernii. There was 2,761 articles for *E. ovinoidalis* in sheep and 2,810 for *E. ninakohlyakimovae* in goats.

## 4. Discussion

Based on the data retrieved, the first publication on anticoccidial drugs was first published in 1949 by Waletzky, Hughes & Brandt on the anticoccidial activity of Nitrophenide (31, 33). Since then, publications on anticoccidial drugs showed progressive advancements. This study’s objective was to evaluate the trends in this field and acknowledge the authors, publications, funding agents, research network in anticoccidial drugs and broad topics that researchers have cited. Based on the performance analysis of publications on anticoccidial drugs, the research established a strong growth in the number of publications and citations over the years. In 2021, there were 393 publications on anticoccidial drugs, which reveals that more and more researchers have continued to gain interest in anticoccidial chemotherapy. Besides, some researchers have found the drug’s efficiency in reducing disease resistance necessitating increased research in the field to unfold the causes and, most importantly, come up with mitigative measures and advanced medicines to fight the pathogens (3, 8, 22). The number of citations in each year has also increased, with a declined trend observed in the last 3 years. This decline can be explained by limited time exposure of the publications to a broad group of researchers and might be due to the closure during COVID-19 outbreak. However, it is highly expected that the total number of citations will increase as more researchers return to pre-COVID-19 circumstances.

The bibliometric analysis identified that despite the anticoccidial drugs made for several animals in the livestock industry, such as dogs, cattle, and rabbits, to name a few, most of the publications were done on chicken (2, 26). Research shows that coccidiosis is ranked among the most costly disease in chickens, explaining the increased studies in the area (27). Besides, the intestinal disease is mainly spread by contact. Among the livestock, poultry is among the most crowded type of farming since several chickens can be reared in a small area (29, 30).

The bibliometric study on anticoccidial drugs is relevant, especially to both veterinary practitioners and scholars, in giving guidance on the trends in the field. Besides, they can gain awareness of the appropriate places to search for relevant information in the field. The co-author network provided, along with other science of mapping visualization techniques, can offer guidance on some of the authors widely known in the field. Besides, other databases can offer more information, such as the most widely used keywords, publications references, and citation networks. Hence, one of the limitations of this study is the use of one database. However, combining several databases may likely lead to biased data since some of the information may be easily duplicated.

The bibliometric analysis supports the importance of pathogenic Eimeria species. According to the estimated publications count, *E. tenella* followed by *E. maxima* and *E. necatrix*. The highly pathogenic chicken Eimeria as *E. tenella* and gut-impacting *E. maxima* are attracting the bulk of research related to coccidiosis. Similarly, *E. bovis* research constituted the major trend in large animals coccidiosis research.

## 5. Conclusion

The number of publications in a field can be taken as a good indicator of its relative level of development. Bibliometrics is the quantitative analysis of the number of scholarly works in a specific field. The existing state of research, its potential for expansion, and the rates at which new publications appear in a field are all things that bibliographic studies are commonly used to assess. As far as we are aware, no studies have been undertaken in these areas; therefore, the purpose of this work is to use bibliometric analysis to compile a large database of articles about anticoccidial medications. Therefore, the current work employs bibliometric analysis to examine key scientific and popular literature in order to trace the development of anticoccidial medications and its effects in the academic and public spheres. Since the first article on anticoccidial medicines appeared in 1949, the research found that there have been three distinct waves of publication and citation activity. During the first phase, which lasted from 1920 to 1968, there were hardly any published studies on anticoccidial medications. The second phase, which lasted from 1969 to 2000, was characterized by a rather stable and slightly elevated number of papers. Between 2002 and 2021, the scientific discipline saw a rising tide of publications and citations. The study detailed the main funding agencies, countries, research institutions, most referenced papers, and significant co-authorship and collaborations involved in the development of anticoccidial medicines. Researchers and veterinarians will benefit from the study’s findings since they will have a better idea of where to look for information about anticoccidial drugs.

## Data availability statement

The original contributions presented in the study are included in the article/Supplementary material, further inquiries can be directed to the corresponding author.

## Author contributions

MK, MAM, HA, MM, MI, MA-R, and HE-B planned the study design, contributed to data analysis, and wrote and revised the manuscript. MK, IA, and KV contributed to data extraction and analysis. All authors contributed to the article and approved the submitted version.

## Funding

This work was supported by the Deanship of Scientific Research, Vice Presidency for Graduate Studies and Scientific Research, King Faisal University, Saudi Arabia (Project# GRANT2865).

## Conflict of interest

The authors declare that the research was conducted in the absence of any commercial or financial relationships that could be construed as a potential conflict of interest.

## Publisher’s note

All claims expressed in this article are solely those of the authors and do not necessarily represent those of their affiliated organizations, or those of the publisher, the editors and the reviewers. Any product that may be evaluated in this article, or claim that may be made by its manufacturer, is not guaranteed or endorsed by the publisher.

## Footnotes

^1^ https://www.dimensions.ai/
